# Implementing a social innovation for community-based peer support for immigrant mothers in Sweden: a mixed-methods process evaluation

**DOI:** 10.3389/fpubh.2023.1332738

**Published:** 2024-01-11

**Authors:** Per Kåks, Linnea Stansert Katzen, Mats Målqvist, Anna Bergström, Sibylle Herzig van Wees

**Affiliations:** ^1^SWEDESD, Department of Women's and Children's Health, Uppsala University, Uppsala, Sweden; ^2^Department of Global Public Health, Karolinska Institute, Stockholm, Sweden

**Keywords:** social innovation, peer support, process evaluation, mixed-methods, parents, children, maternal and child health

## Abstract

**Introduction:**

A South African social innovation based on peer support for mothers was contextualized in southern Sweden. The objective of the project was to support expectant women and mothers of young children in immigrant communities to access public services that would benefit maternal and child health. This study aimed to assess how the intervention was implemented, what the contextual barriers and facilitators were, and how the implementation was perceived by those who delivered and received it.

**Methods:**

The study used mixed methods with a convergent parallel design and followed the Medical Research Council guidance on process evaluations of complex interventions. Semi-structured interviews (*n* = 19) were conducted with peer supporters, client mothers, and key stakeholders involved in the intervention. The qualitative data were analyzed using content analysis. Quantitative data on peer supporters' activities were collected during contacts with client mothers and were presented descriptively.

**Results:**

The five peer supporters had 1,294 contacts with client mothers, of which 507 were first-time contacts. The reach was perceived as wide, and the dose of the intervention was tailored to individual needs. Barriers to implementation included community mistrust of social services, norms on gender roles and parenting, and funding challenges. The implementation was facilitated by the organization's reputation, network, experience, and third-sector affiliation. Peer supporters tended to prioritize linking clients to other services over the educational components of the intervention, sometimes doing more than what was originally planned. Implementation strategies used included building trust, using multiple outreach venues, using internal support structures, and providing practical assistance as an entry point to comprehensive psychosocial support. The personal connection between peer supporters and clients was highly valued, and the building of relationships enabled them to address sensitive topics. Peer supporters sometimes experienced a blurred line between professional and personal roles.

**Conclusions:**

Peer supporters used a variety of strategies to navigate identified barriers and facilitators. Trust was central both as a contextual factor and a strategy for implementation. It is valuable to maintain a balance between flexibility and adherence to the function of peer supporters. Further research is needed to evaluate the effects of the intervention.

## Introduction and aim

In recent decades, Sweden has seen an increasing socioeconomic divide (1). With immigrant families having a higher risk of being on the lower side of the socioeconomic spectrum, this has resulted in inequities in health. One example of this is that infant mortality rates are twice as high for children born to mothers who have immigrated than for those born to Swedish-born mothers (2). Such disparities reflect a complex set of social and health-related factors that can impact the wellbeing of immigrant parents and their children. This includes interactions with the welfare system, such as how different resources and services are used.

Immigrant populations in high-income countries often face multiple challenges in accessing public services. Previous research has indicated that migrants in Europe may find it difficult both to get an overview of what services are available and how they can be accessed (3, 4). When accessing health services, factors such as language difficulties and discrimination can be barriers to their effective use (5). As a consequence, immigrants are less likely than the native population to use preventive services and more likely to use emergency services (5). From a provider's perspective, the key challenges in providing care to immigrant populations in high-income countries have previously been summarized as lack of communication, continuity, and confidence (6).

Qualitative research in Sweden has highlighted how newly arrived immigrant parents struggle to adjust to a new type of social life and a sense of uncertainty surrounding housing, residence permits and income (7, 8). Similar studies have also identified a need for information on what culturally appropriate parenting entails and what support is available from the authorities (9, 10). While parental support groups are offered to all parents in Sweden through the child health services, they are not utilized by immigrant and low-income parents to the same degree as the majority population (11). A report on migration and health in southern Sweden has highlighted lack of trust in institutions as a barrier to welfare consumption, as it is more prevalent among those who have migrated to Sweden than the general population (12). This can sometimes take the form of mistrust of social services, with parents avoiding communicating their support needs due to a fear of being separated from their children on social grounds (9).

Ensuring that the social and health needs of parents who have immigrated to Sweden are met requires new approaches to address how public services are accessed and used. Social innovation has emerged as a conceptual framework to describe the innovative efforts of both civil society, the public sector and parts of the private sector to develop and implement new, efficient solutions to complex societal challenges (13). At the core of the concept is a priority for social rather than commercial benefits. The European Commission has defined social innovation as “new ideas that address social needs, create social relationships and new collaborations” (13), which emphasize the interactive and cooperative aspect of development and implementation. The innovative component can consist of new ways of filling in social gaps by improving access to existing welfare services, and not just the development of new welfare services *per se* (14). Peer support programs are a form of social innovation that has been used to improve health outcomes and integration among marginalized groups (15). The concept is based on the idea that social challenges are best understood from the inside and that those who have successfully navigated such challenges can use their experiences to support others (16).

A variety of social innovations based on peer support targeting marginalized parents have been tested in low-, middle- and high-income countries (15, 17). One such innovation is the Mentor Mother model developed by the Philani Maternal, Child Health and Nutrition Trust in Cape Town, South Africa (18). The Mentor Mother model involves health-focused parenting support provided by women who themselves come from the area where they work as Mentor Mothers. This support is delivered as home visits, provided both in the form of educational interventions and through linking families to clinic-based healthcare and other services when needed. The model is summarized in five principles:

1. A careful recruitment process. The recruitment of Mentor Mothers follows the principle of positive deviance, aiming to identify women who have managed to raise healthy children despite challenging circumstances. This background enables them to act as role models in their work. All mentor mothers work in the area where they live.
2. Appropriate training. The Mentor Mothers initially receive a 6-week course on maternal and child health, motivational interviewing and other intervention strategies. In addition, they also receive continuous training on a monthly basis.
3. Home-based, action-oriented health intervention. The families' homes are used as a platform to discuss the health and wellbeing of children and mothers, focusing on nutrition, HIV, tuberculosis, pregnancy, mental health, personal finances, and more. The Mentor Mother shares her knowledge and skills to help families find their own solutions to the problems they face, rather than solving the problems for them. Visits are made weekly to monthly.
4. In-the-field supervision and support. Each Mentor Mother is regularly supported by coordinators in the field. Time is set aside for debriefing on difficult cases and feedback on performance.
5. Monitoring and performance feedback. Field activities and client health outcomes are tracked over time to enable evaluation of performance and effectiveness. The data also makes it possible to identify particularly vulnerable families.

In 2021, the model was contextualized and implemented as a peer support intervention for immigrant mothers and pregnant women in Malmö in southern Sweden (19). This resulted in a change of focus to social determinants of health, prioritizing linking to other services and new ways of recruiting and maintaining contact with the heterogeneous target group. By evaluating the implementation of this program, we hope to inform both its further development and the development of similar social innovations targeting the health and wellbeing of marginalized communities. We also seek to contribute to the literature on the transfer of social innovations between contexts by illustrating how continuous adaptation and learning can take place within a civil society-led intervention. This study thus aims to contribute to the literature on peer support by increasing the understanding of internal and external factors that influence the possibility of using peer support as a means of promoting health and social inclusion in a high-income context characterized by social divides. The need for such efforts is pressing given the increasing disparities among the Swedish population, which poses unique challenges for the provision of welfare services and public sector interactions with marginalized groups.

### Aim

This article aims to assess how the peer supporters enacted their roles, what adaptations of the social innovation have been made during the course of implementation, how the implementation has been perceived by those who deliver and receive it, and what barriers and facilitators were identified during the implementation process.

## Methods

### Setting

This study was conducted within the organization *Yallatrappan*, a social enterprise based in a socioeconomically disadvantaged area in Malmö. The enterprise focuses on providing platforms for work integration for immigrant women. Since 2021, they also run the peer support program for pregnant immigrant women and mothers of children up to 5 years old. The steering group for the program consists of representatives from Yallatrappan, the Church of Sweden and Uppsala University. It also has a recent addition of representatives from housing companies interested in increasing livelihoods and knowledge of rights and obligations among their tenants.

Malmö is Sweden's third largest city with a population of 357,000, of which 36% are born in a foreign country (20). The city has a high unemployment rate of 12.6% of the population aged 16–64 years, compared to the national average of 6.7% (21). The city is also characterized by a high degree of residential and social segregation, resulting in disparities in health between neighborhoods (22).

### The peer support intervention

The peer support program employs five women from the area, all of whom have themselves immigrated and successfully integrated into Swedish society. Each peer supporter works 30 h a week. They all have previous experience of working with women and children. The peer supporters are selected to cover the most common language groups among immigrants in the area. In their daily work, they conduct outreach to make contact with pregnant women or mothers of children up to 5 years old (subsequently referred to as *client mothers*). They map the client mothers' individual needs regarding contact with public services such as authorities, healthcare or civil society organizations, in order to support the client mothers in these contacts. They also work informatively by educating client mothers about Swedish public and civil society services, as well as on topics related to parenting and maternal and child health. The content of the intervention is largely user-driven and the peer supporters' work is tailored to the individual client mothers' needs.

To guide the implementation of the peer support intervention, a logic model was constructed together with stakeholders comprising the steering group during the first half of 2021. This model described the intended inputs, activities, outputs, outcomes and long-term impact of the intervention, as well as assumed causal mechanisms underpinning the logic model.

**Figure 1:**
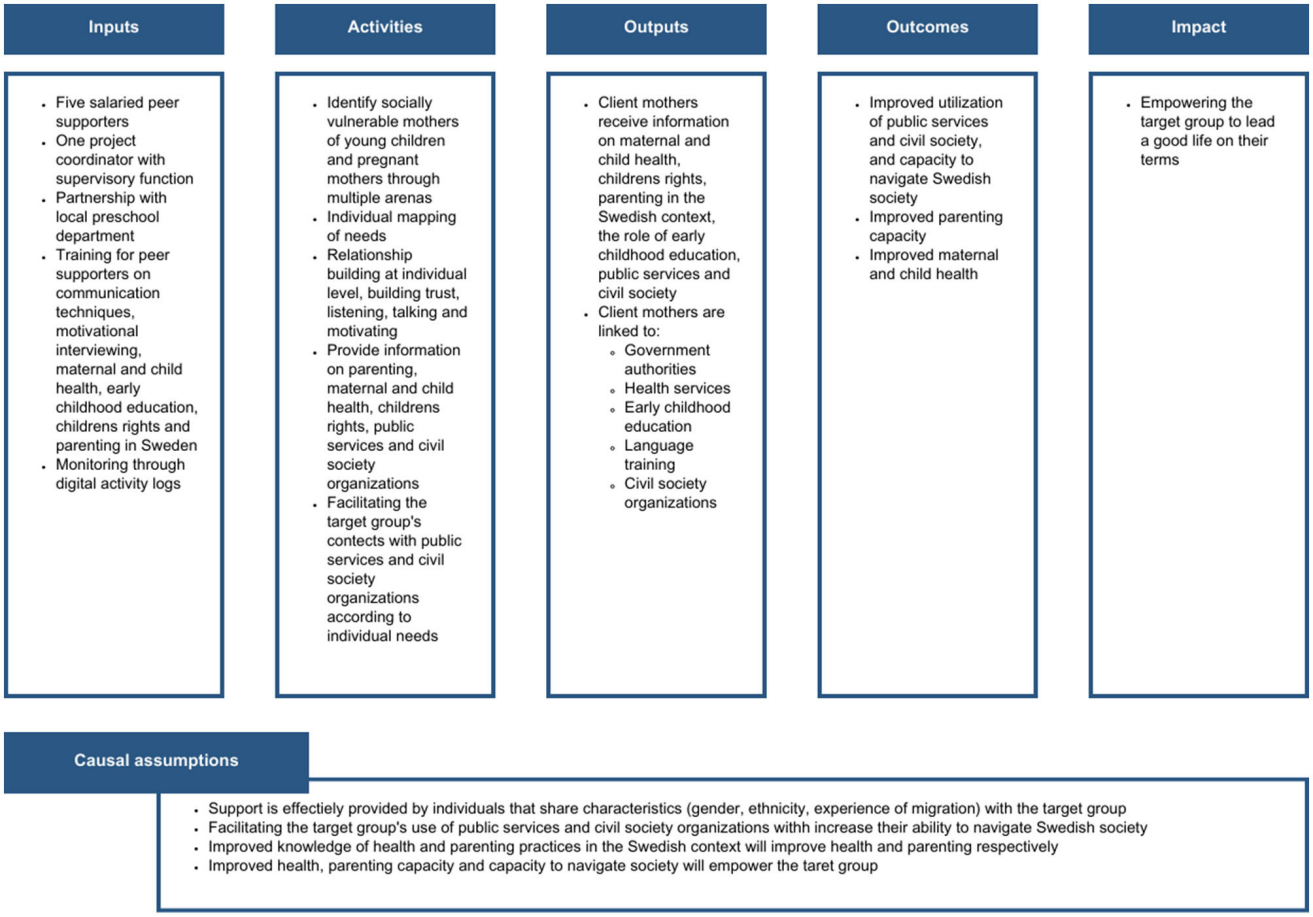
Logic model of the peer support intervention.

As stated in Figure 1, the inputs specified in the model included financing for peer supporters and a coordinator as well as implementation strategies such as partnerships, training, supervision and quality monitoring. The activities included identifying socially vulnerable immigrant mothers and pregnant women in need of assistance in navigating Swedish society and parenting and health practices, and mapping the needs of these women. Subsequently, the peer supporters focus on linking them to public services and civil society organizations based on individual needs. They also support mothers by providing information on how these services operated, and providing information on matters relating to parenting, early childhood education and maternal and child health in the Swedish context. The outputs were defined to correspond to the activities in terms of information received on health, parenting and services, and linking to services and civil society organizations. The outcomes for the intervention were specified to include increased knowledge of Swedish society, citizen rights, parenting and health, and increased use of relevant educational, social and health services, and civil society organizations. The overarching impact was described as empowering of the target group, to contribute to a society where everyone has the possibility to lead a good life.

By clearly describing the components of the logic model, the stakeholders of the peer support project gained a common understanding of what the project was aiming for and how this would be achieved. Furthermore, by defining the activities and outputs, the logic model also enabled an evaluation of the fidelity to these and the identification of possible adaptations within this process evaluation.

### Study design

The study was designed as a mixed-methods process evaluation conducted between January and December 2022. Process evaluations can be used to shed light on the pathways linking the intervention intended to be implemented to the outcomes produced, and has been described as a vital part of community-based interventions (23). The use of both quantitative and qualitative data can offer both opportunities for exploring the findings of one data type using another method, and for determining how different types of data converge or diverge. This study followed a convergent parallel design where qualitative and quantitative data were collected and analyzed simultaneously and integrated at the point of analysis to allow for triangulation (24).

**Figure 2:**
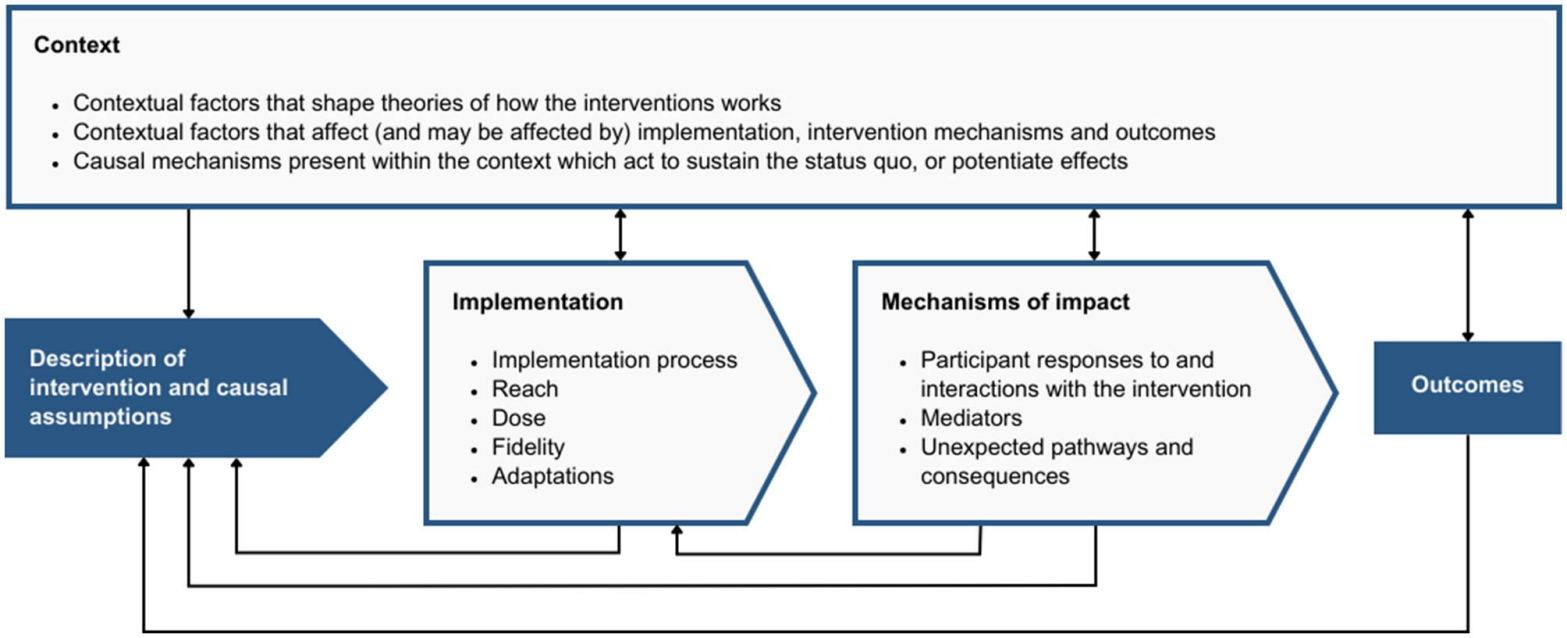
Domains of the MRC framework.

The United Kingdom Medical Research Council (MRC) guidance on process evaluation of complex interventions was used to guide the study (Figure 2) (25). The guidance provides a framework specifying a structured methodology for evaluating key domains that affect the possibility for an intervention to achieve its desired outcomes. These domains include:

- *Context:* external or internal factors affecting the intervention or its implementation,
- *Implementation:* fidelity to the activities specified in the logic model, dose, reach, adaptation and the implementation process, and
- *Mechanisms of impact:* mediating pathways, participant interactions with the intervention and unintended pathways and consequences.

In other words, the MRC framework can be used to investigate what was delivered within the intervention, how it was delivered, the circumstances that have shaped the intervention and its delivery and how the intervention produced change. Understanding these aspects of the implementation process can provide information about how the intervention—and other similar interventions—might be further adapted, improved, scaled, and replicated.

### Data collection

To evaluate the implementation domains of the MRC framework, quantitative data were collected continuously over 12 months, with data collection starting when the intervention had been on-going for 1 year. The data were recorded by peer supporters after each meeting with a client mother, using digital activity logs collected through the software Kobo Toolbox on electronic tablets (26). The data collection forms were constructed in consultation with peer supporters to be comprehensible and feasible to use. The collected data included what information client mothers received during meetings, what type of practical support they received, date and location for meetings, and if it was a physical meeting or through telephone. No data on topics of discussion (i.e., health, parenting, etc.) with client mothers were collected, as this was found to be too complicated to record accurately. To ensure that the implementing civil society organization complied with European Union General Data Protection Regulation (GDPR), no personal data were registered for the client mothers. This also ensured that data collection did not interfere with the intervention logic based on building trust among vulnerable populations. Consequently, it was not possible to follow individuals over time in the log data.

When 6 months of quantitative data collection had passed, qualitative data were gathered from a range of stakeholders involved in the project to explore their experiences of the implementation of the intervention. All five peer supporters employed within the intervention were interviewed in-person on their experiences of delivering the intervention. Digital video interviews were held with all six steering group members (excluding the authors), focusing on the organizational aspects of the implementation process. Eight in-person interviews were also held with client mothers who had received the intervention, to assess how they perceived their need for peer support and how they had received it. They were recruited on the basis that they belonged to two of the main language groups that the peer supporters worked with, Somali and Arabic. The client mothers were recruited by the peer supporters and were interviewed by public health students in their mother tongue. The interview guides used during all interviews were developed to include questions covering the domains of the MRC framework (Supplementary material 1). All nineteen interviewees were female. All interviews were audio recorded and transcribed verbatim, and the interviews held in client mothers' first languages were translated to English or Swedish. Notes on preliminary themes were taken continuously during the process of collecting and transcribing the material.

### Data analysis

The activity log data were compiled and visualized in Tableau 2022.1.0 software (27). The activity log data were divided in terms of if it was the first, second or third or more meeting between peer supporter and client mother. This enabled analyzing how activities changed when they had met several times.

The interview transcripts were imported into NVivo 14 software for data management and analysis (28). The data were analyzed using both deductive and inductive content analysis (29). All transcripts were first coded deductively for manifest content by the first author, using the MRC framework domains as pre-set themes. The data within each sub-domain were then coded again by the first and second author, using an inductive approach to generate codes. The codes were condensed into categories. Repeated reading and re-reading was undertaken to clarify differences, and discrepancies in coding were discussed to generate consensus. The categories were then agreed upon by the first, second, and last author. The generated categories were subsequently discussed within the whole research group before the analysis was finalized.

### Methodological considerations and reflexivity

This study combined the use of qualitative and quantitative data. The advantage of a mixed-methods type of study design is that the research questions can be examined from different angles to increase the validity of the results. The integration of two types of data can be done in different ways. We chose to integrate the data at the point of analysis. An alternative approach could have been to let the quantitative data guide the collection of qualitative data, e.g., by informing the development of interview guides. This could have allowed for a more in-depth exploration of questions raised by the quantitative data, but might also have limited the collection of qualitative data by putting a larger emphasis on exploring quantitative results. It would also have delayed the collection of qualitative data. As the project was in continuous development it was important to capture both qualitative and quantitative data in a synchronous manner.

The interviews with the steering group members and peer supporters were conducted by the first author, who is a white male doctor. The first author had come to know these participants on previous occasions. However, it is possible that the power dynamics of the interview situation influenced what the participants, especially the peer supporters, were comfortable expressing. The interviews may also have been influenced by the fact that it was in the participants' interest to emphasize the positive aspects of the project. However, during the interviews, challenges were repeatedly highlighted and participants were able to problematize aspects of the intervention that had not gone as planned.

The interviews with client mothers were held by two female students fluent in the mothers' respective first languages. No other individuals were present during these interviews, to minimize power discrepancy and promote a free and relaxed interview situation. The client mothers were recruited to the study by the peer supporters. This may have contributed to a social desirability bias, influencing what they said during the interviews and their willingness to criticize the project. The client mothers represented two main language groups in the peer supporters' clientele. The interviews with clients were limited to these two groups as it was necessary to balance the diversity of interviewees with the number of research assistants employed in the study. This choice was also made after discussions with the implementing organization, which saw these language groups as representative of its clientele. However, a greater cultural and linguistic diversity among the interviewees could have contributed to further insights into differences in how the various client groups experienced the intervention.

The quantitative data was collected by the peer supporters themselves. Before the data collection for this study started, the first author and the project coordinator refined the activity log tool over the course of 6 months, continuously consulting the peer supporters. The purpose of this was to develop a tool that measured indicators of importance to the organization, was easy to use and that reliably and consistently captured the activities of the peer supporters. However, a small proportion of activities were not logged immediately after they were carried out, but a week or two afterwards. This may have affected the accuracy of the quantitative data, as the nuances of what was done during the recorded activities may have been lost.

The reliability of the results benefited from the triangulation of methods, sources and analysts. The latter involved double coding of all qualitative data by the first and second authors to ensure a consistent interpretation of the data. The diverse backgrounds of the research team also enabled continuous in-depth discussions with different perspectives on emerging findings before consensus was reached.

### Ethical considerations

The interviews with peer supporters touched on issues of how they succeeded in their role, which was related to their own work performance. The client mothers were interviewed regarding issues of difficulties with integration into Swedish society and how social exclusion affected their ability to provide the best possible care for their children. These areas could be perceived as sensitive, which made it particularly important to explain the purpose and procedures of the study, as well as the confidential handling of the data before they agreed to participate.

Participants were given oral and written information about the study and time to consider their participation before the interviews were held. All participants were also informed that they could withdraw their participation at any time without having to state any reason and without consequences. The voluntary nature of the research project was again emphasized before the interviews began. All participants signed an informed consent form.

The peer supporters were employed within the program and interviews with them were conducted during working hours. Apart from this, the participants received no financial compensation for their part in the study.

## Results

**Figure 3:**
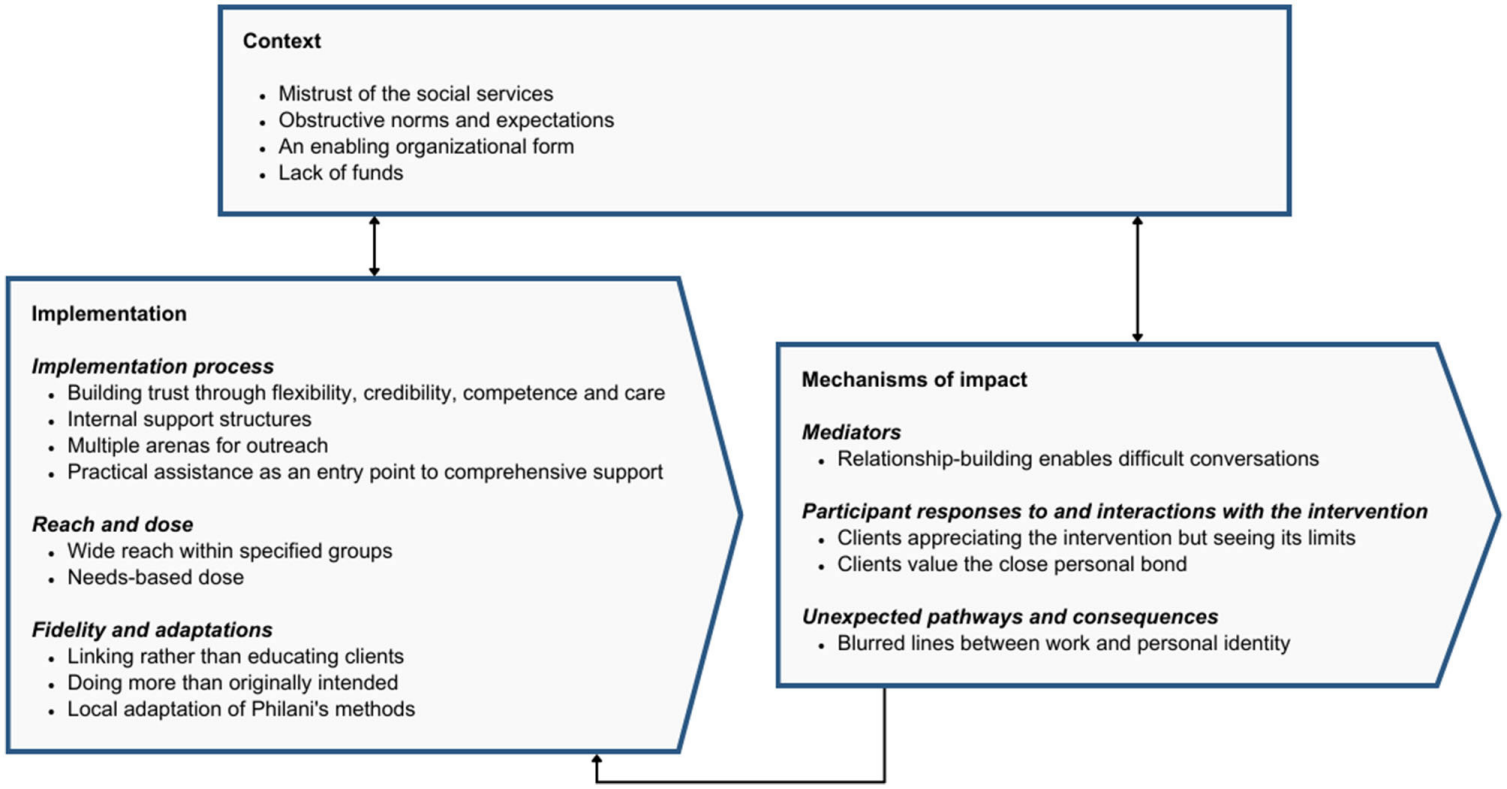
Categories sorted by MRC guidance domains.

Within the domains of the MRC framework, 17 categories were generated in the qualitative analysis, representing different aspects of the context, implementation process, and mechanisms of impact respectively (Figure 3). The categories are illustrated with selected quotes from the interviews and quantitative process data on the peer supporters' activities. Additional quotes are available in Supplementary material 2.

### Context

Several contextual factors were identified both in the internal organizational context and the external context. These constituted both barriers and facilitating factors during implementation.

#### Mistrust of the social services

The peer supporters and steering group members expressed that a common obstacle they had to overcome was a widespread mistrust of social services among the target group. This mistrust stemmed from a knowledge that social services could separate children from their parents on social grounds. Peer supporters described how this led families from the target group to avoid contact with social services. It could also lead them to avoiding contact with other services such as preschools or mental health professionals, based on a fear of being observed by staff who may judge their parenting as inadequate and report them to the social services. Participants described how this avoidance of contact with social services could sometimes lead to families in the target group avoiding contact with peer supports as they perceived them to share certain characteristics with social workers and assumed they were working collaboratively.

*In the beginning, when I talked to someone about that I work as a peer supporter at Yallatrappan and help those in need, they thought I worked with the social services, and they didn't dare talk about anything. They said no, no, no, in case a second mother wanted to say something, the first mother said [hushing sound]. Why, I said. No, no, we have everything, my husband is doing great, my children are doing great. I said wait, you misunderstand. I don't work with the social services [Peer supporter #2]*.

This effect had, however, diminished over time as the peer support intervention became better known among the target group. During the interviews, none of the client mothers brought up any aversive attitudes toward social services or other authorities.

#### Obstructive norms and expectations

Gender norms around domestic responsibilities and autonomy were described in the interviews as resulting in some men being skeptical of their wives' increased engagement in activities outside the home.

*When it comes to something that concerns the woman herself, that she should take a course or that she should go on an outdoor trip or that she should do something for her own sake, she always has to ask her husband. And that's a thing that's quite difficult to work out, like, for the peer supporters to work with, it becomes quite difficult right away, I mean, because you might not want to encourage them to like, go home and ask your husband if you are allowed. You might want to talk about that further, like, remember that you are also an individual [Steering group member #6]*.

Norms around parenting were also discussed with regard to client mothers sometimes wanting their youngest children to stay at home instead of attending preschool. These factors posed challenges that peer supporters had to navigate in their efforts to increase their clients' agency and participation in society.

#### An enabling organizational form

A facilitating factor in the internal, organizational, context that was highlighted during the interviews was Yallatrappan's good reputation. Together with their established network and their history of running large projects, this enabled the establishment of the intervention among both the target group and among the community actors that the peer supporters collaborated with and linked the target group to. The organization's role as a non-governmental organization was also described as providing freedom in the management and development of the intervention and enabling them to act as a neutral part between migrant mothers and the welfare system.

*You only accept information from someone you trust, someone you have a relationship with, someone you have confidence in. It's as simple as that. And that's not always authorities and administrations, but rather us [Yallatrappan], who are more independent in our relationship with the citizens. Plus we have activities that have existed for a long time in the area, that are appreciated and important [Steering group member #2]*.

#### Lack of funds

Participants emphasized that a persistent internal barrier to project implementation was unpredictable funding. This led to difficulties in planning the long-term development of the intervention. It also contributed to difficulties in scaling up the work despite a high perceived need for peer supporters' support. The lack of sustainable funding was discussed as something difficult to understand given the need to work preventively on integration, parenting and health. A factor that was highlighted was that the organization's close cooperation with public actors in other projects had led these actors to prefer to enter into new agreements with other organizations in order to increase their distribution of financing to external projects.

### Implementation

The results related to implementation consisted of reach and dose which were found to vary between groups, and the peer supporters' implementation strategies were considered to consist of both explicit and implicit strategies.

#### Reach and dose

##### Wide reach within specified groups

**Figure 4:**
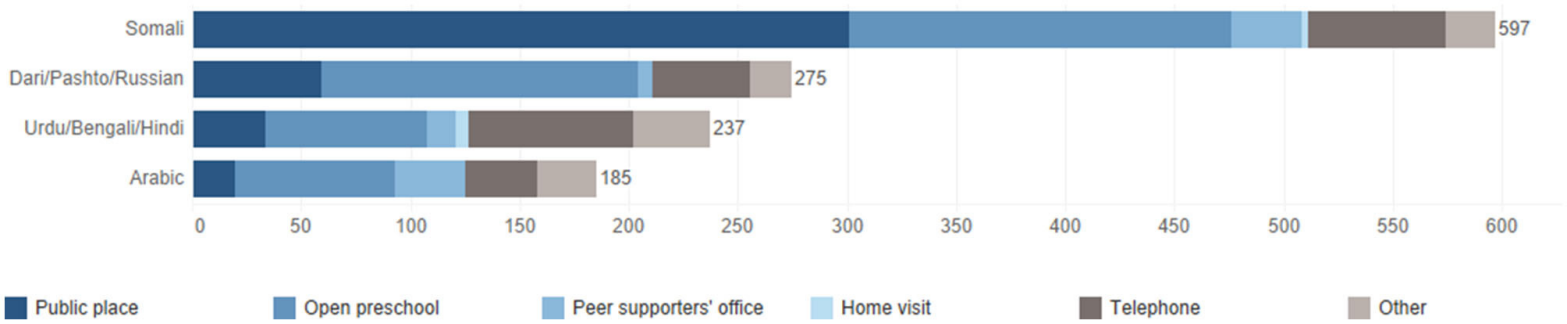
Language groups reached and meeting locations.

A total of 1,294 contacts with client mothers were registered in the activity logs, of which 1,081 (83.5%) were in-person meetings and 213 (16.5%) contacts by phone. Out of the total, 597 (46.1%) contacts with clients were held by two Somali speaking peer supporters, 275 (21.3%) by a peer supporter speaking Dari, Pashto, and Russian, 237 (18.3%) by a peer supporter speaking Urdu, Bengali, and Hindi, and 185 (14.3%) by a peer supporter speaking Arabic (Figure 4).

Overall, peer supporters and steering group members felt that they managed to reach a large number of women in the target area. However, due to the heterogeneous composition of the target group, their coverage was limited to the specific language groups to which they themselves belonged. One language group that fell outside of this was Romani, which participants raised during several interviews.

Another group that was considered difficult for peer supporters to reach was the most isolated women. This was also the group that was perceived to be most in need of the peer supporters' services. One factor in the difficulty of establishing contact with them was the lack of ways to identify them.

*The peer supporters work a lot in their local area where they live, because they have a pretty good idea of who lives around them and so on. But those who are the most isolated, we don't reach them. You probably have to reach them through the social services, and maybe not even then [Steering group member #6]*.

##### Needs-based dose

**Figure 5:**
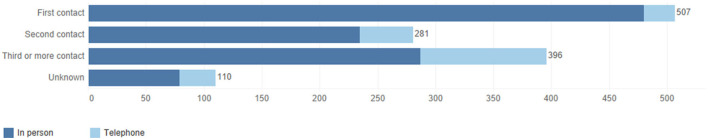
Number of first, second and third or more contacts with client mothers.

The number and frequency of contacts were adapted to individual needs, resulting in a large variation in the amount of contact peer supporters had with individual client mothers. As individual mothers were not followed over time in the log data, the intervention dose could not be quantified. However, peer supporters had about half as many (55.4%) second contacts (*n* = 281) as first contacts (*n* = 507) (Figure 5). This suggested that many client mothers had only a single contact with peer supporters during the data collection period. Peer supporters and client mothers were more likely to talk by phone the longer they had been in contact with each other.

Client mothers voiced that the need for support in practical matters decreased over time, as they became more comfortable with solving the problems they encountered in their daily lives. This meant that contacts with the peer supporter tended to become less frequent with time.

*You see, in the beginning when I first met her, I used to need her [help] a lot with a lot of things. But you know with time, one will get to know things. You become more aware of things. So my need for her now is not like before, but I still need her [Mother #6]*.

#### Fidelity and adaptations

##### Linking rather than educating clients

Peer supporters described that their work had a high adherence to the part of the logic model that involved linking client mothers to other services. However, they tended to focus less on direct intervention in the form of educating mothers in parenting techniques or maternal and child health. This was described as a consequence of not feeling comfortable taking on specialized topics where there were other professionals who had deeper knowledge and could explain things better. Peer supporters also felt it unnecessary to replace existing services where client mothers could receive information, such as parenting courses. In cases where they educated the mothers on issues such as parenting, child health or children's rights, they were careful to clarify the limitations of their own knowledge. The degree to which individual peer supporter focused on educating mothers vs. linking varied from person to person:

*I think some peer supporters [educate mothers] a lot and some peer supporters don't do it at all. I know that [one peer supporter] is very good at doing it, and I've heard her do it too. And she often talks about it, like this, the importance of education. This is very important, this is for the sake of your children. And if your children get a good education, they will be strengthened in life. Very much like that. A lot about gender equality and women's rights and so on. A lot about old cultural structures from [the country of origin] that she talks about in a very nuanced way. While I know that some peer supporters are very practically oriented [Steering group member #6]*.

The log data recorded how the peer supporters worked to link client mothers to different types of services, and whether they provided information about different types of services. In general, peer supporters tended to focus on providing information about open preschools and parenting classes and accompanying mothers to open preschools during the first contacts, while they were more likely to support them in their contacts with the health care providers during later contacts.

##### Doing more than originally intended

Participants stated that the intervention logic model was not used as a tool in their daily work, but rather as a tool to give the project an overall direction. This was related to the flexible design of the intervention, where activities were largely adapted to the expressed needs of the client mothers. In the work of the peer supporters, they stated that they did not always stick to the scope of the project, but often did more for the client mothers than what was in their job description. This could mean helping them with practical matters related to their housing situation or helping them to buy things for the home.

*I helped her a lot with housing. She had no housing. She had a child who had a disability. They lived in a hotel and it was a lot. And the child too, he got even worse with epilepsy, and it was a lot. It affected him. And the social services couldn't help with housing. So I used to help her by going to the housing site here in Malmö, registering for an apartment [Peer supporter #1]*.

It was also sometimes challenging to restrict themselves to the intended target group, partly because it was difficult to refuse support to women without children or mothers with older children when they had explicit needs. This was justified as a potential way of increasing knowledge of the intervention within the target group.

##### Local adaptation of Philani's methods

The evaluation showed that the adaptation of the Philani Mother Mentor model—upon which this intervention was based—resulted in several differences from its original inception. For example, Philani's training material for Mentor Mothers was not used in this project. Instead, other types of training materials in counseling techniques and peer support were used.

During the interviews, the participants also described that the meetings that the peer supporters had with client mothers very rarely took place as home visits, which was in contrast to Philani's philosophy on delivering peer support. Out of the 1,081 physical meetings registered in the activity log data, 9 (0.83%) were conducted in the client mothers' homes (Figure 4). The low use of home visits was partly motivated by the fact that the intervention had started to be implemented during the COVID-19 pandemic, as the routines for the project had been established during a period when home visits were difficult to carry out safely. Peer supporters also expressed that home visits felt too personal and were perceived as time inefficient. An additional factor that was raised was that it could be problematic from a work environment point of view to use the homes for meetings, as it was never possible to know what the environment was like before arriving there. It was also not possible to assume that the client mothers experienced their own home as a safe place for meetings.

#### Implementation process

##### Building trust through flexibility, credibility, competence, and care

The participants described that an important strategy in the implementation of the intervention was to actively work on building trust. This applied both to relations with clients and to relations with other actors who were in contact with the target group, such as maternity and child health centers. The trust with clients was built by the peer supporters, in their role as generalists, by providing a wide range of knowledge about the structure of the Swedish welfare system, parenting, and children's health and wellbeing, while being clear with the clients about the limits of their knowledge. They were also mindful of having a high level of accessibility, being flexible and sensitive to the locations of meetings that client mothers felt safe and suitable, and actively nurturing relationships with their clients. At the organizational level, stakeholders made sure to enable trust building by making careful choices in the recruitment of peer supporters. In some cases, this was achieved by current peer supporters using their own contact networks to find people they thought might be suitable for the role.

*The right people in the right place, yes. Well, that's always what it's all about [Steering group member #2]*.

Their competence was enhanced through continuous training that responds to their perceived needs. Previous training sessions had covered topics such as child development, early childhood education, children's rights, parenting techniques, maternal health, COVID-19 and vaccinations, the organization of the welfare system, housing rights, and psychosocial support techniques. The participants highlighted a need for further training on sexual and reproductive health, handling cases of domestic violence, mental health and stress in the target group, the Swedish legal system, the organization of the social insurance system, and managing aggressive or conflictual behavior in other people.

##### Internal support structures

Another implementation strategy was active supervision with high availability and responsiveness to peer supporters' needs, which was provided through weekly group meetings and continuous contact by phone or through meetings in between set supervision sessions. The peer supporters also described that they also actively helped each other by answering questions from the client mothers and referring them between each other in cases where other language competencies were needed.

##### Multiple arenas for outreach

To reach out to new mothers in need of support, peer supporters used a variety of physical and digital venues. These included spontaneous meetings in public places such as parks or public transport. They could also use venues specifically aimed at the target group, such as open preschools, language courses or meetings at ethnic associations. The digital platforms that enabled contact with new mothers included primarily online groups tied to ethnic associations. The client mothers who took part in the intervention could also pass on the contact within their contact networks, which meant that knowledge of the intervention spread organically to some extent.

Participants also described how staff at child health clinics, maternal health clinics, open preschools and libraries could refer mothers with support needs to them by giving out their phone number or business cards. This was made possible by active networking among these actors to establish knowledge about the intervention which participants described as being effective.

#### Practical assistance as an entry point to comprehensive support

**Figure 6:**
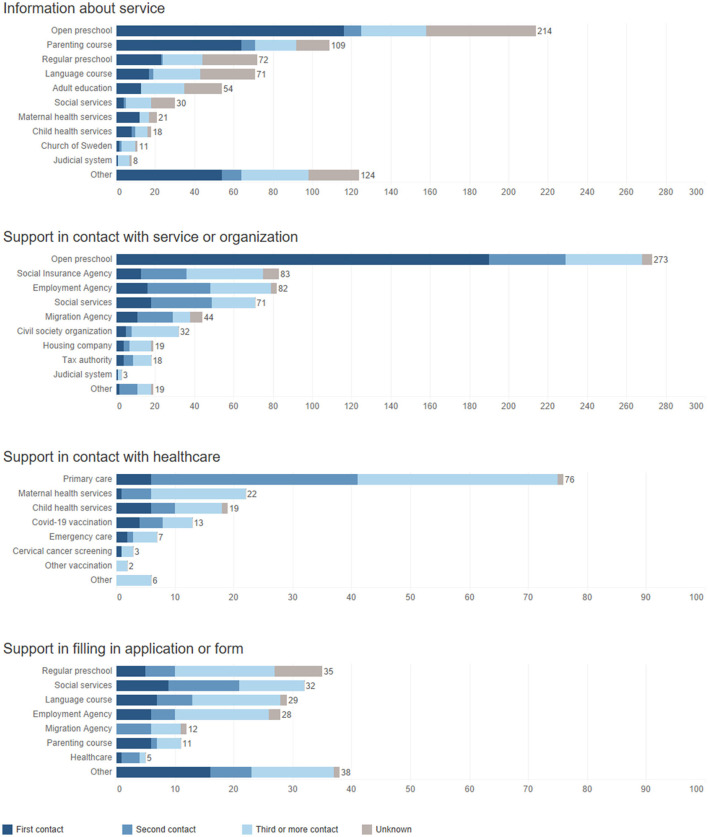
Peer supporters' activities.

One strategy for engaging mothers in the intervention and approaching sensitive topics, described by both peer supporters and steering group members, was to focus on practical support early in the contact with mothers. In practice, this meant that discussions around children's participation in early childhood education was often used as a starting point and first topic of conversation with families. This was also evident in the log data (Figure 6). The focus on early childhood education sometimes meant that peer supporters physically accompanied families to open preschools. During the first conversations with new mothers, peer supporters also raised questions about work, or helped them fill out applications or forms to authorities. These activities were described as undramatic and easy to engage in early on.

Once a good relationship had been established, the peer supporters could start identifying needs that required trust to be discussed. These could be issues of a more sensitive nature, such as psychological or medical needs, or issues related to family relationships or parenting practices.

*It starts with like, can you help me with this paper? There are often very practical concerns to begin with. And then it builds up along the way. [...] So often it's those needs first, and then it's like this... Yes, but knowledge and information. How do you do this? How do you do that? And are you entitled to it and how much does it cost and how do you register, and so on. And one thing often leads to another [Steering group member #6]*.

### Mechanisms of impact

A variety of mechanisms of impact could be identified in the material. These consisted of both mechanisms that facilitated the intervention and constituted secondary effects.

#### Mediators

##### Relationship-building enables difficult conversations

During the interviews, the importance of building relationships with the client mothers was highlighted, as it enabled the peer supporters to gain their trust. A trustful relationship could later open up the possibility to start discussing difficult topics and support them in sensitive matters, e.g., by linking them to health services. In the quantitative data, this could be seen in that contacts with the health services were rarely made during the first meeting, but were more common in later meetings (Figure 6). Relationship building was also described as important to assure mothers of the confidentiality of the conversation.

*It became a beautiful relationship, friendship, not just a formal or limited relationship. [...] This is how this job should be, it shouldn't be that people are just like a train, work, and that's it, their job is done. They have to be friendly, so that people can like them and feel safe and comfortable when they ask for their help. And honestly that's what's happening [Mother #6]*.

#### Participant responses and interactions

##### Clients appreciating the intervention but seeing its limits

The interviews with client mothers highlighted their appreciation of the support they received and the extent of it. Both the practical and psychosocial support they received was described as helping in reducing their everyday worries and anxiety. Some discussed how the intervention helped them to become more independent in their lives, and how they gained a sense of belonging to society.

*When I needed help communicating with the children's schools and I couldn't do it myself, they helped. When I wanted to contact the health service, they taught me how to do it and also helped me with the language. When I had problems with financial assistance, they supported me and talked to the social services officer and explained the situation. As a mother, I could sleep well and my mind calmed down and my worries disappeared. My mind, my soul and my body have become calm. This led me to become more motivated and start thinking about how to develop myself [Mother #3]*.

During the interviews, the limitations of the project were also highlighted, including that the client mothers wished they could have received support in establishing themselves on the labor market.

##### Clients value the close personal bond

In describing the relationship with peer supporters, client mothers tended to describe their relationships with peer supporters in terms of “family” or “friendship.” The close relationship was highlighted as something positive in itself. The informal feel was also described as contributing to the client mothers feeling that there was always a low threshold to contact the peer supporters and that they could discuss anything with them.

*You know I would honestly describe her as a sister, a life-long sister truly. [...] I mean yes, she is a friend, you feel that she is a friend, I mean our relationship is very, very beautiful. You know, she really connects to a person and makes them talk about their matters. This is something that is very comforting to mothers that have come to Malmö recently. They are in need, especially those that do not have anyone, they do not have sisters or relatives here. They need someone close to listen [Mother #7]*.

A contributing factor to this was the shared cultural understanding. This enabled comparisons between the culture in their country of origin and in Sweden and how this related to matters such as different views on parenting.

#### Unexpected pathways and consequences

##### Blurred lines between work and personal identity

In the interviews with peer supporters, they expressed that they often experience a blurred line between work and private life. This could manifest itself in their relationship with the mothers they were in contact with, in that they talked about topics outside the scope of the project, or that they provided support in challenges that were not within the intended scope of their work. Several of the peer supporters described that their activities were more than just work, and that their role had become part of their identity.

The blurred boundary between work and private life could also be expressed by working outside office hours. This phenomenon arose both from the fact that the client mothers could contact them by phone in the evenings and at weekends, the perceived high demand for their services, and the fact that the peer supporters enjoyed their work.

*I work all the time, but I don't feel like it's work. [...] You know when you want to do what you do, you don't feel tired or that it's hard or anything. I don't feel that [Peer supporter #2]*.

In some cases, the mothers were in urgent need of help with contacts with the health service or other agencies outside of office hours. They could, however, also have non-urgent problems that the peer supporters helped them with as they were easy to resolve quickly. The peer supporters also used occasions when they met new mothers during their free time to introduce them to the intervention. The delimitation of working hours was discussed as something that had decreased somewhat the longer the supporters had worked, as they learned to manage their time.

## Discussion

This study aimed to assess how the implementation of a peer support program for immigrant mothers and pregnant women was undertaken and the factors that influenced this process. Our findings highlight that trust was central to both the challenges and the opportunities of the peer support intervention. A critical element in building trustful relationships was a flexible and needs-driven approach to the content of the intervention and how it was delivered. This enabled client mothers to be supported in their interactions with the welfare system, their approach to parenting, and issues relating to health on their own terms. Using this flexibility as a resource in the implementation resulted in an intervention with low fidelity to the educational parts of the program's logic model, where peer supporters tended to focus more on linking their clients to other services.

This study adds to the previous literature on peer support programs aimed at parents. By shedding light on the unique role of peer supporters for migrant parents, it provides information that can inform the development and implementation of this and future interventions. It also contributes to the literature on the transfer of social innovations such as the Philani model between different contexts, by illustrating the value of having an organic approach to local adaptation. Our findings thus link to previous literature on both the role of the peer supporter and debates around flexibility and adaptations of complex interventions.

### Peer supporters' role enactment

Previous research has shown how trust in the social services is lower among socially disadvantaged groups in Sweden (30), which can challenge their perceived legitimacy among the groups that need their services the most. Our findings provide examples of how this lack of trust can have a spill-over effect on parents' readiness to use other services, demonstrating the need for a holistic approach to promoting trust in authorities.

Engaging with client mothers through building trustful relationships constitutes a feature of the peer support intervention that might be difficult to attain within public welfare services, as this process is dependent on an informal, highly personal component. Furthermore, previous research has pointed toward how community-based projects with service providers that share a sociocultural background with their clients enhances engagement through context-sensitive delivery (31). This demonstrates the unique position that peer supporters can have in relation to other professional categories, and the benefits of implementing this type of intervention through a non-governmental organization that is unbound by the constraints and expectations of formality of the public sector. It is also in line with the findings of previous research on peer support for socially disadvantaged mothers, highlighting how it can be easier to open up to peer supporters than public professionals about sensitive topics (32).

Studies on how the third sector interact with hard-to-reach groups have highlighted four factors that contribute to successful engagement: respect and trust, flexibility, collaboration with other services and organizations, and user involvement (33). It is worth noting how these align with how peer supporters used trust building through care and flexibility, a focus on linking to other services and using other services as arenas for recruitment, and adapting to the individual client mothers in terms of what they needed help with, where meetings were held and the number and frequency of contacts.

The addition of new activities described by the study participants reflects the need-driven nature of the intervention, as well as its complexity. Part of the complexity lies in the fact that peer supporters are expected to work as generalists, with consequent difficulties in gaining in-depth knowledge of each topic covered by their role. Holding such a generalist role in the presence of specialists, such as midwives and parenting counselors, raises the threshold for educating the mothers directly and lowers it to focus on referring clients.

The generalist role can also make it more difficult to communicate what the intervention is and what the boundaries of the peer supporters' role is. Previous studies on lay-worker support programs have highlighted how it can be difficult to communicate the function of the generalist lay support worker and how that relate to the functions of other professionals (34). To promote effective use of the service, it is important to provide a clear outline of what the support entails and what it does not.

### Fidelity, adaptations and flexibility with respect to the logic model

Flexibility as a tool for navigating complexity is a critical component of community-based individual support, especially for hard-to-reach groups. The ability to be responsive, creative and holistic constitutes unique contributions of peer supporters in their relationship with a welfare system that is often rigid and fragmented (35).

Individual tailoring to clients' needs and preferences can take different forms, and peer support interventions for parents tend to be flexible in either their content, their mode of delivery or in availability of peer supporters (15). While standardization and careful definition of an intervention is a prerequisite for reliable evaluation of its effects, the inherent aspect of flexibility in complex interventions can sometimes make it difficult to even define what the intervention is, and what it is not (36). Instead of seeing flexibility and individualization of the content as a challenge to rigorous implementation, it can be seen as a core component in itself. This is in line with how core components of parental support programs have been defined in previous studies in Sweden (37). The literature on scaling and transfer of social innovations has described how innovations can be scaled and replicated either with absolute fidelity to a specified plan and program integrity in mind, or with an overall aim of disseminating principles or methods (38). Replicating social innovations, such as the South African Philani model being implemented in Sweden, can thus involve a more complex process than simply diffusing a fixed model. Rather, it can allow for adaptation to emerging circumstances while preserving the essence of the innovation. With this in mind, low fidelity to a program plan is not necessarily a failure of implementation, but an attempt to ensure the relevance of the program in a context of heterogeneous needs. However, it is important that the adaptations made do not compromise the elements or functions that are central to the intervention's internal logic.

The participants in our study described adaptations in the form of the focus on linking to public services and the tendency to do more for client mothers than what was originally specified, e.g., by engaging in housing issues. These adaptations can be outlined and interpreted using the Framework for Reporting Adaptations and Modifications-Expanded (FRAME) (39). This framework provides a systematic approach to characterize modifications made to interventions. It includes specifications of (1) when and how the modification was made, (2) if it was planned or unplanned, (3) who determined that the modifications should be made, (4) what is modified, (5) at what level of delivery the modification is made, (6) the nature of the modification, (7) if it is consistent with fidelity to a program plan, and (8) reasons for modification.

The focus on linking to public services was a shift away from educating mothers directly, which was made during implementation as a reactive change by the individual peer supporters. The modification was made in response to their perception that there were other services that did this better, aiming to improve feasibility. It can be classified as a way of skipping elements of the intervention that might not have been consistent with fidelity to the logic model, as it changed central functions in the intervention logic.

The peer supporters doing more than intended meant the addition of activities such as helping client mothers with housing issues or helping mothers buy specific items. It constituted a modification that was reactive and undertaken by the individual peer supporters in response to emergent circumstances, aiming to improve fit with client mothers, and increase satisfaction. The modification consisted of adding elements in a way that might not have interfered with fidelity to the logic model, as it did not interact with central functions or elements in an apparent way (40).

Adaptations to improve feasibility and fit with recipients have been described as common in previous studies (41, 42). While such changes to the intervention may make it more relevant to its users, it is valuable to aim for planned adaptations rather than reactive modifications. Such a proactive process can improve both engagement and retainment while maintaining the essential aspects of the intervention (42).

In contact with client mothers, the boundaries of the peer supporters' role and tasks were also flexible. Studies of peer support for mothers in South Africa, where the implemented model was originally developed, have highlighted how the work as a Mentor Mother can dissolve the boundary between personal and professional roles in both positive and negative ways (43, 44). This is a phenomenon that has also been seen among lay health workers in other parts of the world (45). The work as a peer supporter can involve feelings of pride in empowering others and a sense of becoming someone with a respected position in the own community. In parallel, the work can also present burdens in terms of expectations of availability and selflessness as strong personal relationships develop with clients. This liminal position, balancing between the formal and the informal, can be described as a central component in the concept of peer support (16).

A review of the mechanisms of successful peer support presented this as the *therapeutic use of the self*, pointing toward how this can promote a sense of responsibility among peer supporters, and the *reframing of identity*, where peer supporters themselves receive a sense of meaning through a reciprocal relationship with their clients (46). With self-identity as an important instrument in their work, it is perhaps not surprising that peer supporters experience blurred boundaries between work and private life. It is, however, important to see this both as a resource and as an issue that needs to be managed through boundary-setting and self-care to maintain feasible and sustainable working conditions (43).

### Implications for practice—Balancing flexibility and standardization

In human-centered design, the success of innovations are considered to rest on three overlapping dimensions: desirability, feasibility and viability (47). Achieving a high degree of all three facilitates the development, implementation and sustainability of the innovation at hand. Using this framework as a lens to reflect on the findings of this study highlights potential implications for future development of the peer support intervention and its implementation.

In the interviews with the clients' mothers, the desirability of peer support was highlighted in various ways. One factor that contributed to the clients' appreciation of the support was its holistic view of their life situation and its ability to help them with a wide variety of problems they encountered, demonstrating the value of not sticking to a delineated project plan with absolute rigidity. To maintain the desirability of the intervention, maintaining the flexibility in the work of the peer supporters is of value.

The feasibility of the peer support intervention reflects the ability of the peer supporters to deliver a high quality intervention also over time. This is partly dependent on clear boundaries for their work in terms of working hours and what their work entails, and their own ability to communicate these boundaries to their clients. The possibility of providing support on sensitive topics was facilitated by relationships built up over time between the peer supporters and their clients. Given that many mothers had only a single contact with peer supporters, it may be worth prioritizing the follow-up of the first contact to enable the identification and addressing of needs that are difficult to discuss in initial meetings.

As highlighted in the interviews with steering group members, the viability of the project was threatened by challenges in securing sustainable funding. While this may reflect a common challenge for third-sector innovations that are dependent on external financing, the chances of sustainable funding may be increased if the benefits of the intervention can be communicated clearly. This in turn is dependent on both clear descriptions of what the intervention is, and outcome evaluations that demonstrate what the eventual effects are, both of which are facilitated by standardization. For complex interventions, this can mean a standardization of processes and functions rather than content (48). In practice, this can translate to consistency in terms of how peer supporters focus on linking or educating mothers respectively rather than consistency in terms of what topics are covered during each meeting with client mothers. Such standardization can permit a flexible approach to meeting the support needs of individual clients, maintaining the holistic model while enabling an intervention that can be clearly communicated and evaluated for effectiveness.

The transferability of the results is limited by the fact that the study was conducted within a specific organization with an intervention that is relatively unique to the Swedish context. However, similar interventions exist in other countries and similar multicultural contexts exist elsewhere in Sweden and internationally. This study's findings can thus inform the development of new interventions and evaluations if they are carefully contextualized.

## Conclusion

In implementing a social innovation for peer support for migrant mothers and pregnant women, the peer supporters faced barriers in the form of mistrust of social services in the community, norms and expectations around the role of women and children, and difficulties with funding. The implementation of the intervention was facilitated by the organization's reputation, network, history of running large projects, and their role as a third-sector organization. The intervention was implemented with a needs-based dose and a perceived good reach within the language groups covered by the peer supporters. Peer supporters prioritized linking their clients to other services, which resulted in an implementation with low fidelity to the educational parts of the intervention, and they tended to do more than what was stated in the intervention description. There were local adaptations of the working methods of the program on which the intervention was based. The implementation process was enabled by the use of strategies to build trust, multiple venues to reach clients, internal support structures such as supervision, training and collaboration between peer supporters, and the use of practical help as a gateway to more comprehensive psychosocial support. Client mothers appreciated the intervention and personal connection with peer supporters, and were able to discuss difficult topics after building deeper relationships with them. Peer supporters could sometimes experience a blurring of the boundaries between professional and private roles. In the future development of this and similar interventions, it is of value to maintain a balance between flexibility of methods and content on the one hand, and clarity regarding the functions and role of peer supporters on the other.

## Data availability statement

The raw data supporting the conclusions of this article will be made available by the authors, without undue reservation.

## Ethics statement

The studies involving humans were approved by the Swedish Ethical Review Authority. The studies were conducted in accordance with the local legislation and institutional requirements. The participants provided their written informed consent to participate in this study.

## Author contributions

PK: Conceptualization, Data curation, Formal analysis, Investigation, Methodology, Project administration, Software, Visualization, Writing—original draft, Writing—review & editing. LS: Formal analysis, Investigation, Methodology, Software, Writing—original draft, Writing—review & editing. MM: Conceptualization, Funding acquisition, Investigation, Methodology, Project administration, Resources, Supervision, Validation, Writing—review & editing. AB: Conceptualization, Investigation, Methodology, Supervision, Validation, Writing—review & editing. SH: Conceptualization, Investigation, Methodology, Supervision, Validation, Writing—review & editing.

## References

[1] Therborn G. Sweden's turn to economic inequality, 1982–2019. Struct Change Econ Dyn. (2020) 52:159–66. 10.1016/j.strueco.2019.10.005

[2] Socialstyrelsen. Socioekonomiska faktorers påverkan på kvinnors och barns hälsa efter förlossning. (2016). Available online at: https://www.socialstyrelsen.se/globalassets/sharepoint-dokument/artikelkatalog/ovrigt/2016-12-14.pdf (accessed May 12, 2023).

[3] Ahmadinia H Eriksson-Backa K Nikou S. Health-seeking behaviours of immigrants, asylum seekers and refugees in Europe: a systematic review of peer-reviewed articles. J Document. (2021) 78:18–41. 10.1108/JD-10-2020-0168

[4] Satinsky E Fuhr DC Woodward A Sondorp E Roberts B. Mental health care utilisation and access among refugees and asylum seekers in Europe: a systematic review. Health Policy. (2019) 123:851–63. 10.1016/j.healthpol.2019.02.007 30850148

[5] Lebano A Hamed S Bradby H Gil-Salmerón A Durá-Ferrandis E Garcés-Ferrer J. Migrants' and refugees' health status and healthcare in Europe: a scoping literature review. BMC Public Health. (2020) 20:1039. 10.1186/s12889-020-08749-8 32605605 PMC7329528

[6] Brandenberger J Tylleskär T Sontag K Peterhans B Ritz N. A systematic literature review of reported challenges in health care delivery to migrants and refugees in high-income countries - the 3C model. BMC Public Health. (2019) 19:755. 10.1186/s12889-019-7049-x 31200684 PMC6567460

[7] Hedstrom E Kovshoff H Hadwin JA. Exploring parenting narratives in asylum seeking populations in Sweden: examining the effect of post-migration stress on families through grounded theory. J Refug Stud. (2021) 34:3381–98. 10.1093/jrs/feaa136

[8] Mangrio E Carlson E Zdravkovic S. Newly arrived refugee parents in Sweden and their experience of the resettlement process: a qualitative study. Scand J Public Health. (2020) 48:699–706. 10.1177/1403494819893535 31841079

[9] Osman F Klingberg-Allvin M Flacking R Schön UK. Parenthood in transition - Somali-born parents' experiences of and needs for parenting support programmes. BMC Int Health Hum Rights. (2016) 16:7. 10.1186/s12914-016-0082-2 26883321 PMC4754847

[10] Herzig van Wees S Fried S Larsson EC. Arabic speaking migrant parents' perceptions of sex education in Sweden: a qualitative study. Sex Reprod Healthc. (2021) 28:100596. 10.1016/j.srhc.2021.100596 33550052

[11] Region Skåne. Föräldrastöd i grupp inom mödra-och barnhälsovården i Skåne [Group parental support in maternal child health care in Scania]. (2017). Available online at: https://vardgivare.skane.se/contentassets/97f0c2e2bb4e42399755032dcf40c060/foraldrastod-i-grupp-inom-modra–och-barnhalsovarden-i-skane-2017.pdf (accessed June 1, 2023).

[12] Mangrio E Carlzén K Grahn M Zdravkovic S. Kartläggning av nyligen nyanländas hälsa, levnadsvanor, sociala relationer, arbetsmarknad och boendemiljö efter etableringen. Delrapport från MILSA 2, 0. (2020).

[13] European Commission. Directorate-General for Internal Market, Industry, Entrepreneurship and SMEs. Social Innovation. European Commission. Available online at: https://single-market-economy.ec.europa.eu/industry/strategy/innovation/social_en (accessed June 1, 2023).

[14] van Niekerk L Manderson L Balabanova D. The application of social innovation in healthcare: a scoping review. Infect Dis Poverty. (2021) 10:26. 10.1186/s40249-021-00794-8 33685487 PMC7938294

[15] Kåks P Målqvist M. Peer support for disadvantaged parents: a narrative review of strategies used in home visiting health interventions in high-income countries. BMC Health Serv Res. (2020) 20:682. 10.1186/s12913-020-05540-8 32703302 PMC7376883

[16] Watson E. The mechanisms underpinning peer support: a literature review. J Ment Health. (2019) 28:677–88. 10.1080/09638237.2017.1417559 29260930

[17] Trickey H. Peer support for breastfeeding continuation: an overview of research. In: Perspective-NCT's journal on preparing parents for birth and early parenthood. (2013), p. 15–20.

[18] Tomlinson M Rotheram-Borus MJ Roux IMl Youssef M Nelson SH Scheffler A. Thirty-six-month outcomes of a generalist paraprofessional perinatal home visiting intervention in South Africa on maternal health and child health and development. Prev Sci. (2016) 17:937–48. 10.1007/s11121-016-0676-x 27438294 PMC5111552

[19] Kåks P Bergström A Herzig van Wees S Målqvist M. Adapting a South African social innovation for maternal peer support to migrant communities in Sweden: a qualitative study. Int J Equity Health. (2022) 21:88. 10.1186/s12939-022-01687-4 35733169 PMC9217115

[20] Statistics Sweden. Inrikes och utrikes födda efter region, ålder och kön. År 2000–2022 [Domestic and foreign born by region, age and gender. Year 2000–2022]. Statistikdatabasen [Statistics database]. Available online at: https://www.statistikdatabasen.scb.se/pxweb/sv/ssd/START__BE__BE0101__BE0101E/InrUtrFoddaRegAlKon/ (accessed April 8, 2023).

[21] City of Malmö. Occupation. City of Malmö: Facts and statistics in English (2020). Available online at: https://malmo.se/Facts-and-statistics/Occupation.html (accessed April 8, 2023).

[22] Commission for a Socially Sustainable Malmö. Malmö's path towards a sustainable future: Health, welfare and justice. (2013). Available online at: https://malmo.se/download/18,6c.44cd5c1728328333211d32/1593519743583/malmo%CC%88kommisionen_rapport_engelsk_web.pdf (accessed April 8, 2023).

[23] Skivington K Matthews L Simpson SA Craig P Baird J Blazeby JM. A new framework for developing and evaluating complex interventions: update of Medical Research Council guidance. BMJ. (2021) 374:n2061. 10.1136/bmj.n2061 34593508 PMC8482308

[24] Schoonenboom J Johnson RB. How to construct a mixed methods research design. Kolner Z Soz Sozpsychol. (2017) 69:107–31. 10.1007/s11577-017-0454-1 28989188 PMC5602001

[25] Moore GF Audrey S Barker M Bond L Bonell C Hardeman W. Process evaluation of complex interventions: medical research council guidance. BMJ. (2015) 350:h1258. 10.1136/bmj.h1258 25791983 PMC4366184

[26] KoboToolbox. Kobo. Available online at: https://www.kobotoolbox.org/ (accessed May 12, 2023).

[27] Salesforce. Tableau 2022.1.0. Tableau Software. Available online at: https://www.tableau.com/ (accessed May 3, 2023).

[28] Lumivero. NVivo. (2023).

[29] Elo S Kyngäs H. The qualitative content analysis process. J Adv Nurs. (2008) 62:107–15. 10.1111/j.1365-2648.2007.04569.x 18352969

[30] Nilsson K Landstedt E. Public trust of social workers in Sweden: a repeated cross-sectional study. J Soc Work. (2022) 22:1374–93. 10.1177/14680173221094535

[31] Lawlor ER Cupples ME Donnelly M Tully MA. Implementing community-based health promotion in socio-economically disadvantaged areas: a qualitative study. J Public Health. (2020) 42:839–47. 10.1093/pubmed/fdz167 31822896 PMC7685857

[32] McLeish J Redshaw M. Mothers' accounts of the impact on emotional wellbeing of organised peer support in pregnancy and early parenthood: a qualitative study. BMC Pregn Childbirth. (2017) 17:28. 10.1186/s12884-017-1220-0 28086827 PMC5237175

[33] Flanagan SM Hancock B. ‘Reaching the hard to reach'–lessons learned from the VCS (voluntary and community Sector). A qualitative study. BMC Health Serv Res. (2010) 10:92. 10.1186/1472-6963-10-92 20377850 PMC2856561

[34] Murphy CA Cupples ME Percy A Halliday HL Stewart MC. Peer-mentoring for first-time mothers from areas of socio-economic disadvantage: a qualitative study within a randomised controlled trial. BMC Health Serv Res. (2008) 8:46. 10.1186/1472-6963-8-46 18304334 PMC2291460

[35] Ingram M Reinschmidt KM Schachter KA Davidson CL Sabo SJ De Zapien JG. Establishing a professional profile of community health workers: results from a national study of roles, activities and training. J Commun Health. (2012) 37:529–537. 10.1007/s10900-011-9475-2 21964912 PMC6684283

[36] Datta J Petticrew M. Challenges to evaluating complex interventions: a content analysis of published papers. BMC Public Health. (2013) 13:568. 10.1186/1471-2458-13-568 23758638 PMC3699389

[37] Barboza M Marttila A Burström B Kulane A. Towards health equity: core components of an extended home visiting intervention in disadvantaged areas of Sweden. BMC Public Health. (2022) 22:1091. 10.1186/s12889-022-13492-3 35650586 PMC9158140

[38] Riddell D Moore ML. Scaling out, scaling up, scaling deep. McConnell Foundation. JW McConnell Family Foundation & Tamarack Institute (2015).

[39] Wiltsey Stirman S Baumann AA Miller CJ. The FRAME: an expanded framework for reporting adaptations and modifications to evidence-based interventions. Implement Sci. (2019) 14:58. 10.1186/s13012-019-0898-y 31171014 PMC6554895

[40] Carroll C Patterson M Wood S Booth A Rick J Balain SA. conceptual framework for implementation fidelity. Implement Sci. (2007) 2:40. 10.1186/1748-5908-2-40 18053122 PMC2213686

[41] Strehlenert H Hedberg Rundgren E Sjunnestrand M Hasson H. Fidelity to and adaptation of evidence-based interventions in the social work literature: a scoping review. Br J Soc Work. (2023) 17:bcad170. 10.1093/bjsw/bcad170

[42] Bromley AR. Flexibility within fidelity: a narrative review of practitioner modifications to child welfare interventions. Child Youth Serv Rev. (2023) 149:106908. 10.1016/j.childyouth.2023.106908

[43] Laurenzi CA Skeen S Rabie S Coetzee BJ Notholi V Bishop J. Balancing roles and blurring boundaries: Community health workers' experiences of navigating the crossroads between personal and professional life in rural South Africa. Health Soc Care Commun. (2021) 29:1249–59. 10.1111/hsc.13153 32885519

[44] Katzen LS Skeen S Dippenaar E Laurenzi C Notholi V Roux Kl. Are we listening to community health workers? Experiences of the community health worker journey in rural South Africa. Res Nurs Health. (2022) 45:380–9. 10.1002/nur.22220 35184308 PMC9271365

[45] Glenton C Colvin CJ Carlsen B Swartz A Lewin S Noyes J. Barriers and facilitators to the implementation of lay health worker programmes to improve access to maternal and child health: a qualitative evidence synthesis. Cochrane Database Syst Rev. (2013) 2013:CD010414. 10.1002/14651858.CD010414 24101553 PMC6396344

[46] MacLellan J Surey J Abubakar I Stagg HR. Peer support workers in health: a qualitative metasynthesis of their experiences. PLoS ONE. (2015) 10:e0141122. 10.1371/journal.pone.0141122 26516780 PMC4627791

[47] Chasanidou D Gasparini AA Lee E. Design thinking methods and tools for innovation. In: Design, User Experience, and Usability: Design Discourse. Springer International Publishing (2015), p. 12–23. 10.1007/978-3-319-20886-2_2

[48] Hawe P Shiell A Riley T. Complex interventions: how ‘out of control' can a randomised controlled trial be? BMJ. (2004) 328:1561–3. 10.1136/bmj.328.7455.1561 15217878 PMC437159

