# Supplementary material for: Implementing a social innovation for community-based peer support for immigrant mothers in Sweden: a mixed-methods process evaluation
Source: Front Public Health. 2024 Jan 11;11:1332738. doi: 10.3389/fpubh.2023.1332738 (PMC10821792; doi:10.3389/fpubh.2023.1332738)
Supplement: Supplementary file 1 [file Data_Sheet_1.docx]

## Interview guide - Client mothers

**Introduction**

The interviewer goes through the following before the actual interview and audio recording begins:

- Presentation of the interviewer
- Short presentation of what the interview will focus on
- Explain anonymity and confidentiality
- Explain the recording, the length of the interview (approx. 30-60 minutes), the interview format and the output of the study
- Review consent to participate in the interview and their right to withdraw or skip questions at any time
- Ensure that the informed consent form is signed
- Any questions from the interviewee before starting
- Check that interviewee is comfortable with recording

**Interview questions**

- How did you make contact with the peer supporter?
  - Did you contact the peer supporter or did the peer supporter contact you?
  - Where did you hear about the peer supporters? Where did you get the information?
  - What made you want to make contact? Why did you want support from the peer supporter?
- Can you tell us about what you have done when you have met or had contact by phone?
  - Can you give an example of such a meeting or telephone contact - how did it happen?
  - What do you usually talk about when you are in contact?
  - How do you feel the meetings have gone?
  - How has the peer supporter supported you?
  - Why was it valuable to have support for that particular issue?
  - What have the meetings with the pilot led to? What happened next?
  - Has there been anything that you would have liked to have received help with that was not addressed during the contacts with the peer supporter?
  - Has there been anything that you wanted help with, but that the peer supporter was unable or unwilling to help you with? What was the reason for this?
  - How often do you meet, talk on the phone or text?
- How would you describe your relationship with the peer supporter?
  - Has your relationship changed since you first met? In what way?
- Can you tell us what has been valuable when you have been in contact with the peer supporter?
  - Why was it valuable?
- Is there anything that has been difficult when dealing with the peer supporter?
  - Why has it been difficult?
- Can you tell us what could have been better?
  - Why should it be improved?
- How do you think the work of peer supporters should change in the future?

**Conclusion**

- Is there anything that you think has been important in your contact with the pilot that we haven't talked about?
- Do you have any questions?

The interviewer thanks you for the interview and repeats the information about anonymity and confidentiality.

# Interview guide - Peer supporters

**Introduction**

The interviewer goes through the following before the actual interview and audio recording begins:

- Presentation of the interviewer
- Short presentation of what the interview will focus on
- Explain anonymity and confidentiality
- Explain the recording, the length of the interview (approx. 30-60 minutes), the interview format and the output of the study
- Review consent to participate in the interview and their right to withdraw or skip questions at any time
- Ensure that the informed consent form is signed
- Any questions from the interviewee before starting
- Check that interviewee is comfortable with recording

**Interview questions**

- How did you become a peer supporter?
- Why did you want to become a peer supporter?
- How has the training taken place?
- What does a normal work day look like?
- How do you think the daily work is going? At the open preschools and when you meet families?
- How do the supervision of peer supporters work?
- How do you find/make contact with new families?
- Which families usually want help?
- What do they usually want help with? Why?
- Which families tend to be difficult to reach? If so, why?
- How many new families do you usually get in touch with each week/month?
- How do meetings with families usually take place?
- What do you usually talk about/do when you meet?
- What do the meetings usually lead to in the long run? Do you have an example?
- How many times do you usually meet with each family?
- Do you have an example of a meeting where you were able to help a family?
  - What was it that made it go well?
  - Has it led to anything concrete?
- How has it been possible to work with the thematic areas?
  - Linking to other services and actors
  - Educating client mothers on parenting and maternal and child health
  - Inspiring motivation to participate actively in society
- What do you find difficult in your work?
  - Do you have an example of a meeting where it was difficult to help a family?
  - What was it that made it difficult?
- How often do families come back to the open preschools?
- Have you changed your working methods since you started working?
  - What is it that has made you work differently than before?
- How do you think the work of peer supporters should change in the future?

**Conclusion**

- Is there anything that you think has been important in your contact with the pilot that we haven't talked about?
- Do you have any questions?

The interviewer thanks you for the interview and repeats the information about anonymity and confidentiality.

## Interview guide - Steering committee

**Introduction**

The interviewer goes through the following before the actual interview and audio recording begins:

- Presentation of the interviewer
- Short presentation of what the interview will focus on
- Explain anonymity and confidentiality
- Explain the recording, the length of the interview (approx. 30-60 minutes), the interview format and the output of the study
- Review consent to participate in the interview and their right to withdraw or skip questions at any time
- Ensure that the informed consent form is signed
- Any questions from the interviewee before starting
- Check that interviewee is comfortable with recording

**Interview questions**

- How has the peer support project worked over the last year?
  - For the peer supporters in the field?
  - At the organizational level?
- How was the recruitment of new peer supporters done?
- What effects have you seen from the peer supporters' work?
- How have the peer supporters worked to achieve this?
- Have you received any feedback from families?
- Have you made any changes to the way peer supporters work?
- To what degree has it been possible to follow the logic model?
- Which objectives of the peer supporters' work have been achieved?
  - Why those objectives?
- Which objectives have been difficult to work towards?
  - If so, why?
  - How have you dealt with this?
- Is there anything that has been challenging in the peer support project?
  - If so, why?
- How have you dealt with it?
- Is there anything in particular that has been a key to success over the past year?
  - Has it changed the way you work?
- What role has material from Philani played in the pilot project?
  - Has it changed the work in any way?
- How has supervision been provided?
  - What have the peer supporters needed support in?
- How have the peer supporters been trained?
  - Has the training changed over time?

**Conclusion**

- Is there anything that you think has been important in your contact with the pilot that we haven't talked about?
- Do you have any questions?

The interviewer thanks you for the interview and repeats the information about anonymity and confidentiality.
